# Avian *Plasmodium* in invasive and native mosquitoes from southern Spain

**DOI:** 10.1186/s13071-024-06133-8

**Published:** 2024-01-29

**Authors:** Marta Garrigós, Jesús Veiga, Mario Garrido, Clotilde Marín, Jesús Recuero, María José Rosales, Manuel Morales-Yuste, Josué Martínez-de la Puente

**Affiliations:** 1 Doñana Biological Station, EBD-CSIC, Seville, Spain; 2 Department of Parasitology, University of Granada, Granada, Spain; 3 Veterinary and Conservation Department, Bioparc Fuengirola, Malaga, Spain; 4 CIBER Epidemiology and Public Health (CIBERESP), Madrid, Spain

**Keywords:** *Aedes albopictus*, Alien species, Asian tiger mosquito, Avian malaria, Birds, *Culex pipiens*, *Culiseta longiareolata*, Vector-borne diseases

## Abstract

**Background:**

The emergence of diseases of public health concern is enhanced by factors associated with global change, such as the introduction of invasive species. The Asian tiger mosquito (*Aedes albopictus*), considered a competent vector of different viruses and parasites, has been successfully introduced into Europe in recent decades. Molecular screening of parasites in mosquitoes (i.e. molecular xenomonitoring) is essential to understand the potential role of different native and invasive mosquito species in the local circulation of vector-borne parasites affecting both humans and wildlife.

**Methods:**

The presence of avian *Plasmodium* parasites was molecularly tested in mosquitoes trapped in five localities with different environmental characteristics in southern Spain from May to November 2022. The species analyzed included the native *Culex pipiens* and *Culiseta longiareolata* and the invasive *Ae. albopictus*.

**Results:**

Avian *Plasmodium* DNA was only found in *Cx. pipiens* with 31 positive out of 165 mosquito pools tested. None of the *Ae. albopictus* or *Cs. longiareolata* pools were positive for avian malaria parasites. Overall, eight *Plasmodium* lineages were identified, including a new lineage described here. No significant differences in parasite prevalence were found between localities or sampling sessions.

**Conclusions:**

Unlike the invasive *Ae. albopictus*, *Cx. pipiens* plays a key role in the transmission of avian *Plasmodium* in southern Spain. However, due to the recent establishment of *Ae. albopictus* in the area, further research on the role of this species in the local transmission of vector-borne pathogens with different reservoirs is required.

**Graphical Abstract:**

**Figure:**
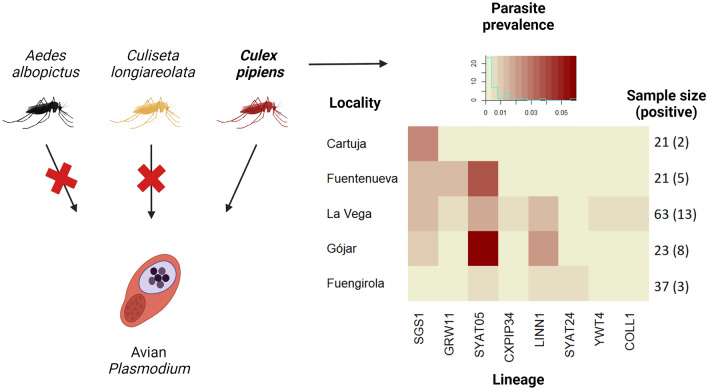

**Supplementary Information:**

The online version contains supplementary material available at 10.1186/s13071-024-06133-8.

## Background

Factors associated with global change, including climate change, habitat alteration and the introduction of invasive species, have largely contributed to the emergence of diseases of public health relevance [1]. Some mosquito-borne pathogens causing diseases such as malaria and West Nile fever are major health concerns nowadays, causing human fatalities worldwide [2]. Different mosquito species have been introduced during the last decades in areas where they were not present before, potentially altering the transmission dynamics of vector-borne pathogens affecting humans and wildlife. In addition, the costs derived for the management of invasive mosquito populations and the damage that they may produce are relevant from an economic perspective [3].

The genus *Aedes* includes different mosquito species with a clear invasive character. Among them, the Asian tiger mosquito *Aedes albopictus* is a widespread mosquito species catalogued as one of the 100 most invasive species according to the Invasive Species Specialist Group [4]. Native from southeast Asia, this species has spread to different areas around the globe in the last decades [5]. In 1979, Albania was the first area where *Ae. albopictus* was detected in Europe [6] but, nowadays, this species is widely present in the continent. In Spain, *Ae. albopictus* was first identified in Catalonia and has progressively colonized areas to southern Spain [7]. *Aedes albopictus* represents a major public health concern as a competent vector of viruses, such as dengue virus, Zika virus and chikungunya virus, and parasites including *Dirofilaria* [8–12].

Avian malaria parasites are widespread haemosporidians of the genus *Plasmodium* naturally circulating between birds and mosquitoes. Endemic avian malaria produces deleterious effects on birds, affecting their health status, survival probability and reproductive success [13, 14]. A recent study in the UK also supported the role of avian malaria parasites as a major factor explaining the population decline of a common bird species, the house sparrow *Passer domesticus* [15]. More relevant is the detrimental effect of invasive avian *Plasmodium* parasites on native immunologically naïve host species [16]. For example, the introduction of avian *Plasmodium relictum* together with its vector *Culex quinquefasciatus* strongly contributed to the population decline of native bird species in Hawaii [16–19]. However, despite their importance for the transmission of avian *Plasmodium*, the study of avian malaria parasites has been mainly focused on the vertebrate hosts, while the role of mosquitoes has been comparatively less investigated.

The role of recently established invasive mosquito species as vectors of avian malaria is poorly studied. *Aedes albopictus* uses mammals as the main bloodmeal sources, although birds represent about 5–10% of its diet [20]. Avian species including *Passer montanus*, *Turdus merula*, and *Gallus domesticus*, among others, are known hosts of *Ae. albopictus* in the invaded areas [20–22], suggesting a potential role of this vector in the transmission of avian *Plasmodium*. This is further supported by laboratory studies where *Plasmodium gallinaceum*, *P. fallax*, and *P. lophurae* completed their cycle in *Ae. albopictus* [23–25], although with lower probability than in other mosquitoes species [18]. In field studies, the presence of avian *Plasmodium* DNA in *Ae. albopictus* has been reported in Japan [26] and in engorged mosquito females from Italy [21]. In a previous study in northeastern Spain, none of the *Ae. albopictus* pools tested positive for avian *Plasmodium,* while 4 out of 167 pools containing 1190 female *Culex pipiens* harbored parasites [21]. These studies suggest that some discrepancies exist regarding the contribution of *Ae. albopictus* in avian *Plasmodium* transmission in nature. Contrary to *Ae. albopictus*, the role of certain native species in Spain to transmit avian *Plasmodium* has been proven [23]*. Culex pipiens* mostly feeds on birds [21, 27], and the molecular screening of avian malaria parasites in mosquitoes revealed the key role of this species as vectors of avian *Plasmodium* in Europe [28, 29]. Furthermore, some studies have proven experimentally the capacity of *Cx. pipiens* to transmit different *Plasmodium* species [23, 30–32]. On the other hand, *Culiseta longiareolata* is considered a bird-bitten species [33], and its role as an avian *Plasmodium* vector has also been demonstrated experimentally [34].

Here, a molecular xenomonitoring approach was used to explore the potential role of the invasive *Ae. albopictus* compared with two common native mosquito species in the local transmission of avian malaria parasites of the genus *Plasmodium* in the recently invaded area of southern Spain. To do that, field-collected mosquitoes were used to molecularly identify the presence of avian *Plasmodium* in mosquito females grouped in pools and to estimate its prevalence in the mosquito populations, also testing the potential temporal and spatial variation on avian *Plasmodium* prevalence in mosquitoes in southern Spain.

## Methods

### Study area and mosquito sampling

**Fig. 1:**
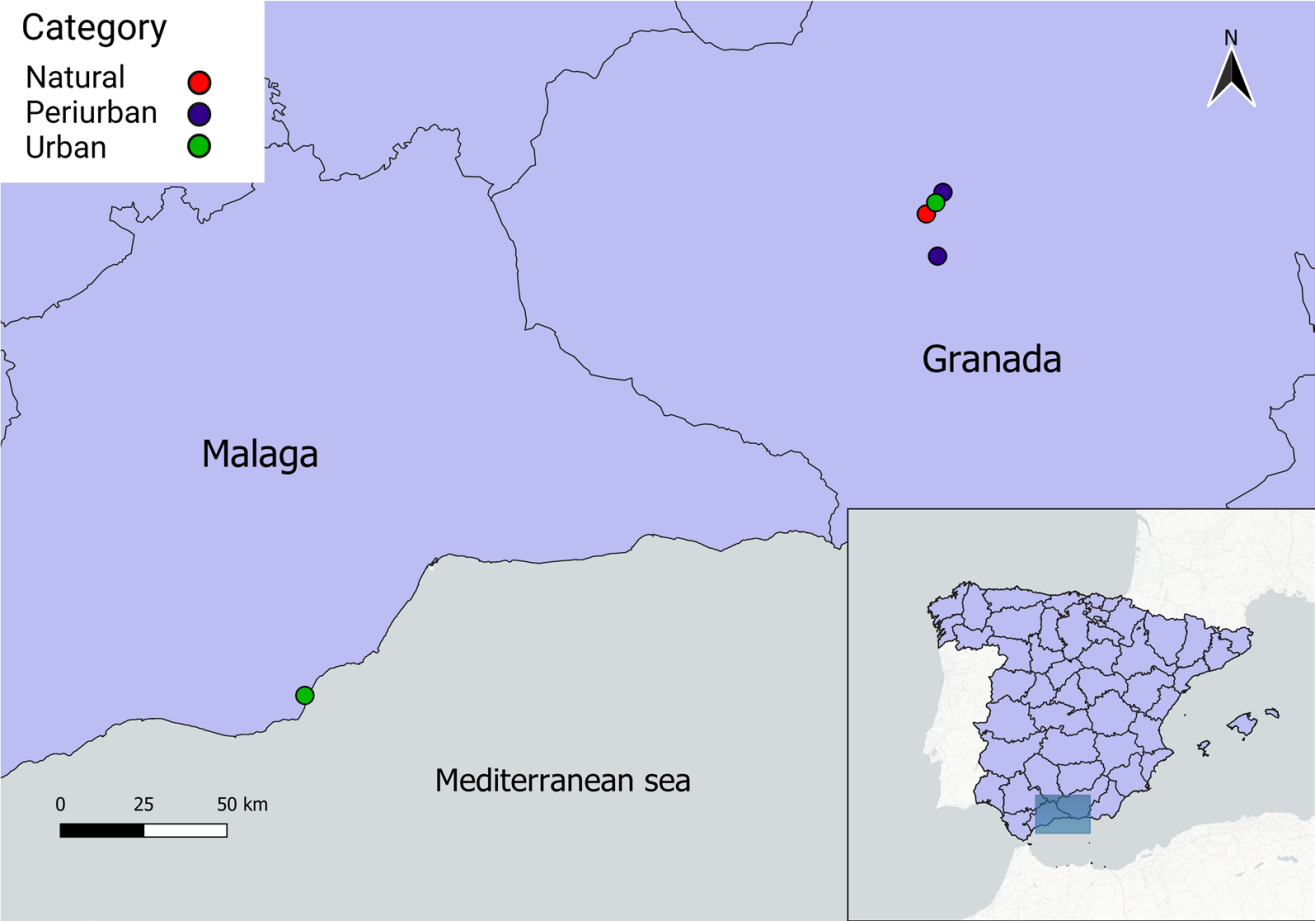
Study area with the five sampled localities in the provinces of Granada (upper right points) and Malaga (lower left point) in southern Spain. Point colors correspond to the habitat category of each locality

Mosquitoes were trapped from May to November 2022 in five localities of the provinces of Granada (*n* = 4) and Malaga (*n* = 1), southern Spain. Sampling sites included two urban sites, two peri-urban sites and one natural site, which differ in terms of land use and human density (see Additional file 1). Specifically, the four sampling sites in Granada included: one natural area sited in a sewage station surrounded by agriculture fields (La Vega, 37°09′57.6"N; 3°37′27.6"W), one urban area in the Fuentenueva campus of the University of Granada (UGR) (37°10′50.4"N; 3°36′32.7"W) and two periurban areas in the Cartuja campus of the UGR (37°11′38.7"N; 3°35′51.2"W) and Gójar (37°06′40.2"N; 3°36′22.4"W) (Fig. 1). In addition, mosquitoes were collected in the urban site of the Bioparc zoological garden (Fuengirola, Malaga province, 36°32′16.7"N; 4°37′39.5"W) to identify the role of mosquitoes in the transmission of avian malaria parasites in an area with a high diversity of wildlife species. Habitat categories were assigned based on visual inspection of the areas and subsequently confirmed according to GIS analyses in QGIS v3.18.1 [35]. Details of this procedure are shown in Additional file 1.

In each locality, trapping sessions were conducted every 2–3 weeks for 24 h approximately, except for the zoo, where traps operated from 7:00 p.m. to 10:00 a.m. (local time) to avoid the influence of visitors. Mosquitoes were collected with four traps per locality and sampling session, two Blacklight (UV)-CDC Miniature traps (Centers for Disease Control and Prevention, Atlanta, GA, USA) and two Biogents (BG)-Sentinel-2 traps (Biogents, Regensbourg, Germany), the last supplemented with dry ice as a source of CO_2_ and BG-Lure mosquito attractant. In each locality, two subzones were established, separated by approximately 10 to 50 m. In each subzone, one Blacklight (UV)-CDC Miniature trap and one Biogents (BG)-sentinel-2 trap were placed nearby. One additional CDC-UV trap placed in a third subzone was used in the zoological garden to maximize the number of mosquito captured in this area. Mosquitoes were frozen and transported to the laboratory on dry ice, where they were identified morphologically using available keys [35, 36] and maintained frozen (– 80ºC) until further molecular analyses. Mosquitoes without any apparent rest of blood meal in their abdomen corresponding to the same species, trapping session and locality were pooled in independent Eppendorfs. Each mosquito pool included the same number of mosquitoes (4 mosquito females) to avoid the potential effect of pool size on the probability of parasite detection.

### Molecular analyses

Genomic DNA from each mosquito pool containing four mosquito females each was extracted using the DNeasy ^®^ Blood & Tissue kit (Qiagen, Hilden, Germany). Molecular amplifications of *Plasmodium* DNA were conducted using the protocol developed by Hellgren et al. [37].  At least one negative control per 20 samples was included during DNA extractions and one negative control per plate for PCR reactions. Each sample was screened twice to avoid false-negative samples [38]. The presence of amplicons was verified in 2% agarose gels. Positive samples were sequenced in both directions using the facilities of STAB-VIDA (Lisbon, Portugal). Sequences were edited using Geneious v2023.2.1 and parasite lineages were identified and named using BLASTN (Basic Local Alignment Search Tool) with sequences deposited in GenBank and MalAvi [39] databases.

### Statistical analyses

The dataset analyzed here includes information from 204 mosquito pools of three different species: *Cx. pipiens* (*n* = 165), *Ae. albopictus* (*n* = 23) and *Cs. longiareolata* (*n* = 16).

Two different analyses were conducted. First, a Fisher’s exact test was used to identify interspecific differences in avian *Plasmodium* infection frequencies among the three mosquito species. Subsequently, comparisons between pairs of mosquito species were tested adjusting *P*-values for type I error and applying the Bonferroni method. Second, a generalized linear model (GLM) with a binomial distributed error and a ‘logit’ link function was fitted to test whether locality and sampling session affected the parasite prevalence in *Cx. pipiens* pools. Analyses were restricted to *Cx. pipiens* pools because parasites were only detected in this species (see results). The model included the avian *Plasmodium* infection status (binary; 1: infected, 0: uninfected) as the dependent variable, and sampling locality (factor with 5 levels) and the sampling session (continuous) as independent variables. The sampling session was included in the model because the prevalence of avian *Plasmodium* is expected to increase in mosquitoes in southern Spain from spring to autumn [28]. The model also included the interaction between sampling locality and sampling session, as seasonal effects might be associated with local abiotic and biotic characteristics. A backward stepwise model selection procedure was performed, starting with the most complex model that included locality, sampling session and their interaction as explanatory variables. Next, the variables that presented a non-significant coefficient were excluded, from the highest to the lowest *P*-value. Statistical analyses were run in R using the packages *stats* [40] and *rstatix* [41]. Parasite prevalence for mosquito species was estimated using the perfect test and exact confidence limits according to pool size in Epitools (https://epitools.ausvet.com.au/).

## Results

**Fig. 2:**
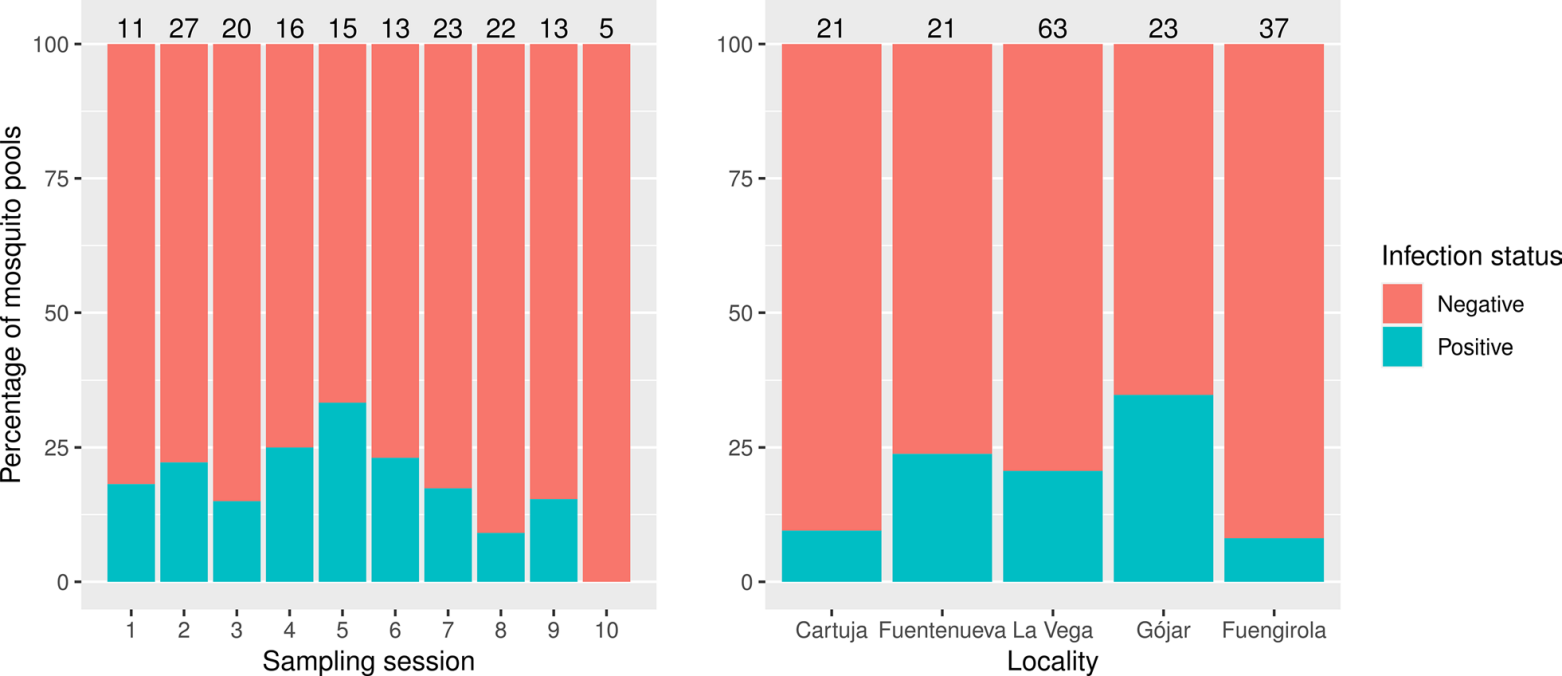
Percentage of *Culex pipiens* pools (y axis) that tested positive or negative for avian *Plasmodium* according to both sampling session (left) and locality (right). Numbers above the bars indicate the sample size for each sampling session/locality. Mosquitoes were trapped from May to November 2022, with the sampling sessions regularly distributed during this period

A total of 204 pools containing 816 mosquitoes were analyzed including the species *Cx. pipiens* (*n* = 165 mosquito pools), *Ae. albopictus* (*n* = 23 mosquito pools) and *Cs. longiareolata* (n = 16 mosquito pools). Thirty-one pools were positive for the presence of avian malaria parasites, all of them corresponding to *Cx. pipiens*. The Fisher’s exact test further supported the significant differences in the prevalence of infection among mosquito species (*P* = 0.007). The pairwise comparisons showed significant differences in the parasite prevalence between *Cx. pipiens* and *Ae. albopictus* (*P* = 0.049) but not between *Cx. pipiens* and *Cs. longiareolata* (*P* = 0.110) and between *Ae. albopictus* and *Cs. longiareolata* (*P* = 1.000). The estimated prevalence of infection in *Cx. pipiens* using Epitools was 0.051 (C.I._95%_: 0.035–0.072). Further GLM analysis on *Cx. pipiens* pools revealed non-significant effects for sampling locality, sampling session and their interaction on parasite prevalence, with none of these variables being retained in the final model (all *P* > 0.050) (Fig. 2).

**Fig. 3:**
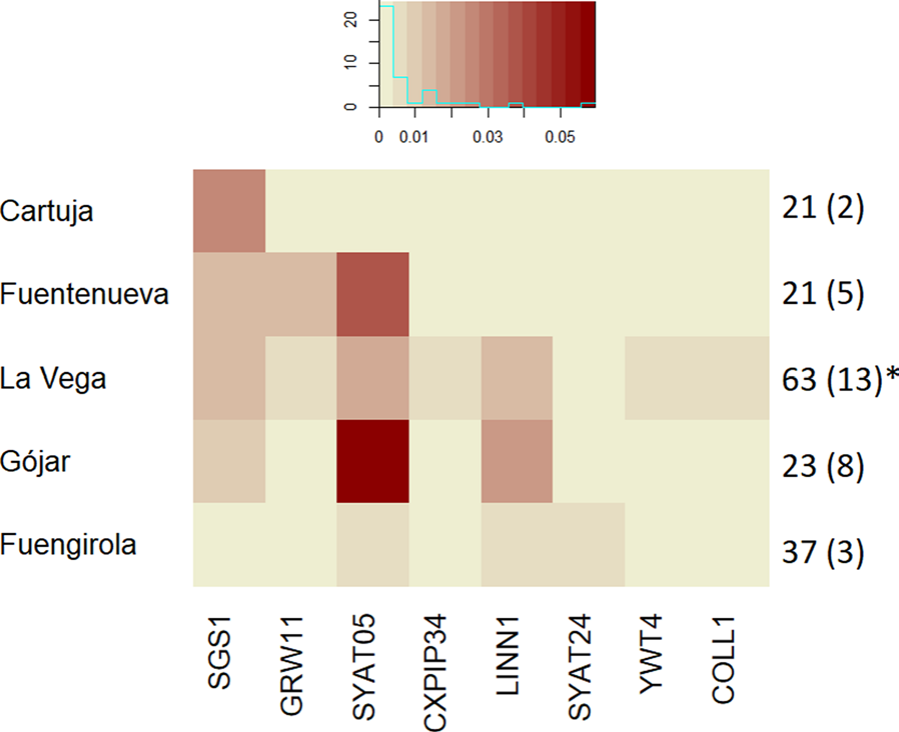
Heat map of the prevalence of infection by different lineages of avian *Plasmodium* parasites (x axis) found in *Cx. pipiens* pools from five localities (y axis) in the provinces of Granada (Cartuja, Fuentenueva, La Vega and Gójar) and Málaga (Fuengirola). The text on the right represents the total number of *Cx. pipiens* pools tested and those positive for avian *Plasmodium* between brackets. *One pool was coinfected with the lineages SYAT05 and SGS1. The legend on the top indicates the match between color and prevalence, with a darker red as prevalence increases (x-axis), while the histogram represents the number of lineages (y-axis) exhibiting a given prevalence (in blue)

Avian *Plasmodium* was detected in *Cx. pipiens* pools from all the sampling localities, although the identity of lineages and their prevalence differed among areas (Fig. 3). Overall, eight *Plasmodium* lineages were found in *Cx. pipiens* pools (Fig. 3), including *Plasmodium vaughani* SYAT05 (*n* = 13), *P. relictum* SGS1 (*n* = 7) and GRW11 (*n* = 2), *P. matutinum* LINN1 (*n* = 6) and *Plasmodium* sp. SYAT24 (*n* = 1), YWT4 (*n* = 1) and COLL1 (*n* = 1). Furthermore, a new lineage of *Plasmodium* sp. was found and named CXPIP34 (*n* = 1; GenBank reference: OR587915). Of them, the lineages SYAT05 and SGS1 were tentatively identified in a single *Cx. pipiens* pool (parasite coinfection) based on the identification of the double peaks in the chromatogram. *Plasmodium* lineages found in the different localities range from one in the periurban area of Cartuja to seven in the natural area of La Vega. Lineages SGS1 and SYAT05 were found in four of the five sampling localities, while the lineage LINN1 was found in three localities. Each of the lineages SYAT24, YWT4, COLL1 and CXPIP34 was only found in a single locality (Fig. 3).

## Discussion

An extensive molecular screening of avian *Plasmodium* in wild native and invasive mosquitoes from two provinces of southern Spain was conducted. The local circulation of avian malaria parasites in the area was confirmed, with the species *Cx. pipiens* likely playing a central role in their transmission. Different lineages of avian *Plasmodium* belonging to at least three different morphospecies were recorded in this area. Contrary to the case of *Cx. pipiens*, further support for the apparent absence of avian *Plasmodium* in *Ae. albopictus* was provided, potentially supporting the low relevance of this invasive species in the local transmission of avian malaria parasites in the area.

### Differences between mosquito species

*Culex pipiens* is an ornithophilic species with birds representing 69–97% of its diet [42]. This species frequently feeds on *Plasmodium*-infected birds, interacting with avian blood parasites under natural conditions [27]. Different laboratory studies have identified the competence of this species for the transmission of avian malaria parasites, including studies using different lineages and species of *Plasmodium* [23]. For example, the presence of parasite DNA in the saliva of *Cx. pipiens* mosquitoes exposed to *P. relictum*- and *Plasmodium cathemerium*-infected birds was previously confirmed [30, 43, 44]. Similarly, experimental studies using lineages such as *P. relictum* SGS1, GRW4 and GRW11 [31, 32, 45] and *P. cathemerium* PADOM02 [46] also confirmed the competence of *Cx. pipiens* mosquitoes for their transmission. In the wild, molecular screening of parasites in mosquito pools identified the presence of avian *Plasmodium* in *Cx. pipiens* from different European countries, including Spain [28, 47, 48]. Particularly in southern Spain, the prevalence of avian *Plasmodium* parasites has been found to range between 2–3.2% in *Cx. pipiens* [28, 49], a slightly lower value than that found in this study (5.1%). Overall, our results add support for the key role of *Cx. pipiens* mosquitoes in the transmission of avian *Plasmodium* under natural conditions.

The statistical analysis supported the difference in parasite prevalence between mosquito species, with a significantly higher prevalence found in *Cx. pipiens* than in *Ae. albopictus*. A similar trend was found between *Cx. pipiens* and *Cs. longiareolata*, although the lack of significance when comparing these two species is probably due to the relatively low number of *Cs. longiareolata* pools analyzed here, as a total absence of avian *Plasmodium* was found in *Ae. albopictus* and *Cs. longiareolata* pools. Although both species are able to feed on birds, potentially affecting the transmission of avian *Plasmodium*, mammals clearly dominate the diet of *Ae. albopictus* [20, 27, 49]. This mammal-bias feeding pattern may explain the low contribution of this invasive species to the transmission of avian blood parasites. In a previous study in the Minami Daito Island of Japan, authors identified the presence of avian *Plasmodium* DNA in a single pool of *Ae. albopictus* out of the 46 mosquito pools tested containing 81 mosquitoes [26]. However, in accordance with our results, additional parasite screenings in *Ae. albopictus* from Spain and Japan reported the absence of avian *Plasmodium* [50, 56]. The low susceptibility of *Ae. albopictus* to avian malaria could also account for our results. LaPointe et al. [18] reported that even when avian *Plasmodium* could complete sporogony in both *Cx. quinquefasciatus* and *Ae. albopictus*, their susceptibility differs greatly, reducing the probability of parasite detection in *Ae. albopictus*. Thus, both the low exposure to avian blood parasites and a reduced competence for the development of parasites in mosquitoes may explain, at least in part, the absence of *Plasmodium* found in *Ae. albopictus*. However, this may not be the case for *Cs. longiareolata*, which is known as a common bird-bitten species [27]. In laboratory studies, avian malaria parasites completed their development in *Cs. longiareolata* females [23]. Nevertheless, to our knowledge, there are no laboratory studies on the interaction between *Cs. longiareolata* and the avian *Plasmodium* lineages circulating in southern Spain, parasites which could be unable to complete their cycle in this mosquito species. Indeed, for the case of field-collected mosquitoes in other regions of Spain, previous studies revealed a total absence of avian *Plasmodium* in *Cs. longiareolata* [50, 51]. However, Mora-Rubio et al. [51] found DNA of the related *Haemoproteus* parasites, which may be mainly transmitted by other insect groups (i.e. *Culicoides*) [52]. Thus, although the small sample size for this species could partly explain the total absence of avian *Plasmodium*, current evidence supports the limited epidemiological relevance of *Cs. longiareolata* in this geographical area for the transmission of avian malaria parasites. Furthermore, the lower relative abundance of *Ae. albopictus* and *Cs. longiareolata* in relation with *Cx pipiens* could also help explain its lower epidemiological importance. In addition, since traps in the zoological garden operated during a shorter period than in the rest of the localities, our sampling size in this area could be affected. However, this fact is not expected to affect the conclusions obtained based on the extremely low prevalence of avian *Plasmodium* found in these species here and in other previous studies [50, 51].

### Lack of effect of locality and seasonality

No significant differences were found in the prevalence of infection in *Cx. pipiens* according to the sampling locality and sampling session. This supports a similar prevalence of infections between areas despite their diverse landscape characteristics (see Additional file 1) and likely host community composition, which may alter the epidemiology of parasites circulating in the area [53]. Sampling mosquitoes in more localities with different characteristics could result in the identification of spatial differences in parasite prevalence in *Cx. pipiens*. However, in line with our results, *Cx. pipiens* show variable avian malaria prevalence in different geographical areas [28, 54, 55], with non-significant differences among sampling points or localities in the same region [56–58]. However, some differences were found in relation to the parasite lineages circulating in the different areas, with some lineages (e.g. SGS1 and SYAT05) being recorded in most of the sampling localities while four lineages were only found in a single locality. A different composition of bird and/or mosquito communities in these areas may explain these differences [53]. Interestingly, three lineages were found circulating in the zoological garden, which have been previously recorded infecting different bird species in southern Spain including common blackbirds [39], a common species in this locality (authors personal observation). The circulation of these lineages in the zoological garden should be relevant from a veterinary perspective, as they have been previously associated with mortality events of immunologically naïve species such as penguins [59, 60]. On the other hand, several studies showed a variation in the prevalence of avian *Plasmodium* infection in *Cx. pipiens* mosquitoes according to season [58, 61, 62], one of them also conducted in southern Spain [28]. In this case, the maximum peak of infection prevalence was reached in autumn [28, 51], which has been attributed, among other factors, to the increased abundance of immunological naïve bird individuals (yearling birds) [28, 63–65]. The lack of differences found here could be partly due to the fact that most of the mosquito samples were taken in summer, while comparatively a lower number of samples from spring and autumn were included in the analyses. In addition, one limitation of our study is that mosquitoes were sampled during a single year. Although similar procedures have been used in other previous studies [28, 51], interannual differences in factors such as temperature may largely determine the community of vectors [66] and, potentially, the local circulation of mosquito-borne parasites [67].

## Conclusions

*Culex pipiens* mosquito females play a central role in the transmission of avian *Plasmodium* in southern Spain. By contrast, our results suggest that the invasive *Ae. albopictus* might play a minor role as vectors of these pathogens, probably due to differences in their blood feeding patterns, pathogen susceptibility and relative abundance. Further studies analyzing additional mosquitoes from other areas and years should be carried out to confirm the low relevance of this invasive species in the local circulation of avian pathogens. Due to the similarities in the epidemiology of avian *Plasmodium* with other pathogens which use birds as reservoirs and mosquitoes as vectors, such as the zoonotic West Nile virus [68], our results could shed light on the epidemiology of pathogens with public health relevance. This is especially relevant considering the local circulation of WNV in southern Spain [69], a zoonotic pathogen occasionally transmitted by mosquitoes from birds to humans, horses and other vertebrates [70].

### Supplementary Information

**Additional file 1**: Detailed description of GIS Analysis and **Table S1 :** Habitat characteristics of the five localities included in this study.

## Data Availability

The data supporting the findings of the study must be available within the article and/or its supplementary materials, or deposited in a publicly available database.
