# Correction: Avian *Plasmodium* in invasive and native mosquitoes from southern Spain

**DOI:** 10.1186/s13071-024-06228-2

**Published:** 2024-03-15

**Authors:** Marta Garrigós, Jesús Veiga, Mario Garrido, Clotilde Marín, Jesús Recuero, María José Rosales, Manuel Morales-Yuste, Josué Martínez-de la Puente

**Affiliations:** 1 Doñana Biological Station, EBD-CSIC, Seville, Spain; 2 Department of Parasitology, University of Granada, Granada, Spain; 3 Veterinary and Conservation Department, Bioparc Fuengirola, Malaga, Spain; 4 CIBER Epidemiology and Public Health (CIBERESP), Madrid, Spain

**Correction: Parasites & Vectors (2024) 17:40** 10.1186/s13071-024-06133-8

Following publication of the original article [1], the authors flagged that they had erroneously omitted the following acknowledgement of Funding from the declaration of their article: Finally, this paper was financed by Programa QUALIFICA, Junta de Andalucía, Consejería de Universidad, Investigación e Innovación (project QUAL21020 EBD). The declaration has since been corrected. The authors thank you for reading this erratum and apologize for any inconvenience caused.

## References

[1] Garrigós M, Veiga J, Garrido M, Marín C, Recuero J, Rosales MJ, Morales-Yuste M, Martínez-de la Puente J (2024). Avian *Plasmodium* in invasive and native mosquitoes from southern Spain. Parasit Vectors.

