# Supplementary material for: Avian Plasmodium in invasive and native mosquitoes from southern Spain
Source: Parasit Vectors. 2024 Jan 29;17:40. doi: 10.1186/s13071-024-06133-8 (PMC10826103; doi:10.1186/s13071-024-06133-8)
Supplement: Supplementary file 1 — Additional file 1: Detailed description of GIS Analysis and Table S1 : Habitat characteristics of the five localities included in this study. [file 13071_2024_6133_MOESM1_ESM.docx]

**Additional file**

*GIS analyses*

Information about land use and population density was obtained from <http://www.juntadeandalucia.es/institutodeestadisticaycartografia/DERA/>. We set up buffers of 500 meters radius around each sampling point to quantify the land use in each buffer by using the 'disolve' and 'intersect' geoprocessing tools, and then the 'statistics by categories' tool, obtaining the total area and the percentage of each variable per buffer. For the land use, we obtained a total of 8 categories grouped into 2 classes. We considered as natural areas the categories: "permanently irrigated land", "mainly agricultural land, but including natural vegetation", "olive groves", "crop mosaic", and "natural grasslands" while urban areas included "industrial or commercial zones", "continuous urban fabric", and "discontinuous urban fabric" categories. In addition, for the same buffer, we estimated the number of people living around sampling sites by using most updated available information (2021 census) of the Institute of Statistics and Cartography of Andalusia. This information is available for a grid of 250 × 250 m and we used the 'intersect' geoprocessing tool to determine the percentage of each grid within each buffer and used the 'statistics by categories' to estimate the total population in these grids.

**Table S1.** Habitat characteristics of the five localities included in this study.

| **Site** | **Province** | **Category** | **Percentage of urban area** | **Percentage of natural area** | **Human density (people/km^2^)** |
| --- | --- | --- | --- | --- | --- |
| Cartuja | Granada | Periurban | 21 | 79 | 587 |
| Fuentenueva | Granada | Urban | 0 | 100 | 7666 |
| La Vega | Granada | Natural | 100 | 0 | 5 |
| Gójar | Granada | Periurban | 55 | 45 | 924 |
| Fuengirola | Malaga | Urban | 0 | 100 | 15045 |
